# Traumatic stress symptoms and PTSD risk in children served by Children’s Advocacy Centers

**DOI:** 10.3389/fpsyt.2023.1202085

**Published:** 2023-06-29

**Authors:** Elizabeth A. McGuier, Kristine A. Campbell, Kara A. Byrne, Lindsay D. Shepard, Brooks R. Keeshin

**Affiliations:** ^1^Department of Psychiatry, University of Pittsburgh School of Medicine, Pittsburgh, PA, United States; ^2^Department of Pediatrics, Center for Safe and Healthy Families, University of Utah, Salt Lake City, UT, United States

**Keywords:** child abuse, posttraumatic stress disorder (PTSD), traumatic stress, children’s advocacy centers, screening, care process model for pediatric traumatic stress

## Abstract

**Purpose:**

Children who experience maltreatment are at high risk for posttraumatic stress disorder (PTSD). Children’s Advocacy Centers (CACs) can facilitate access to treatment following maltreatment allegations. We describe PTSD symptoms and intervention decision-making for children served by CACs.

**Methods:**

Children served by CACs in a single state were screened for PTSD symptoms using a structured mental health screening/referral protocol. CAC staff used an electronic form that provided guidance for decision-making. We examined descriptive statistics for PTSD symptoms and risk and tested associations between child characteristics and symptoms. We described CAC staff’s delivery of brief interventions and referral decisions and tested associations with child characteristics and symptoms.

**Results:**

Two thousand and three hundred fifty children completed screening between 2018 and 2020. Almost half (45.5%) exhibited traumatic stress symptoms suggesting high probability of PTSD at the time of their CAC visit. Children who identified as female or transgender male and older children were more likely to be at high risk for PTSD. Brief interventions were delivered to 66% of children, and most were referred to evidence-based trauma treatment (53.1%) or community mental health services (39.0%). Categorization as moderate or high PTSD risk was associated with a higher likelihood of brief intervention delivery and referral to trauma treatment.

**Conclusion:**

Many children served by CACs are likely to meet criteria for PTSD at their initial visit. CAC staff demonstrated the ability to deliver brief interventions and make referrals to mental health treatment. Use of structured screening/referral protocols may improve early identification and treatment access for children experiencing PTSD symptoms.

## Introduction

Child maltreatment and associated mental health problems are critical public health concerns (1–3). At least 1 in 8 and as many as 1 in 3 children experience maltreatment in their lifetime (4, 5), placing them at elevated risk for posttraumatic stress disorder (PTSD) and other mental health problems (6–9). Sexual abuse specifically is associated with more than twice the risk of PTSD and more than three times the risk of suicide attempts (6, 10).

Children’s Advocacy Centers (CACs) provide coordinated interagency investigations and services after allegations of sexual abuse and other serious maltreatment (11–14). CACs are well-positioned to identify children with mental health needs and facilitate access to mental health assessment and treatment (15).

In 2018, CACs across the state of Utah began implementing a structured protocol to screen and respond to children at risk for traumatic stress symptoms and suicidality at the time of their CAC visit. This protocol, the Care Process Model for Pediatric Traumatic Stress (CPM-PTS), provides a road map of care and electronic screening and decision support tools to assist frontline staff in screening for and responding to traumatic stress symptoms and suicidality (16). Screening for traumatic stress and referrals to evidence-based trauma treatments are critical components of trauma-informed systems of care (17). Development, implementation, and use of the CPM-PTS are described by Byrne et al. (18). In this brief report, we present results from the first 2 years of CPM-PTS use in Utah CACs. We describe the prevalence of traumatic stress symptoms and PTSD risk among children served by CACs, test associations between child characteristics and symptoms, and describe CAC staff decision-making for children reporting elevated symptoms.

## Methods

### Setting and procedures

The current project is an observational study of the prevalence of traumatic stress symptoms in children seen in CACs in Utah between March 2018 and February 2020. Data were available from 16 CACs that implemented the CPM-PTS; four CACs were in urban areas and the rest in rural/frontier areas (19). In most CACs, the CPM-PTS was administered by staff without clinical training (12/16; 75%), most often victim advocates. The CPM-PTS was intended for use with all children between 5 and 18 years old. Child symptoms and staff decision-making were collected through two tools built into the HIPAA-compliant, web-based Research Electronic Data Capture (REDCap) platform hosted by the University of Utah (20, 21). Two REDCap tools supported CPM-PTS administration: a client/family-facing trauma screening (Pediatric Traumatic Stress Screening Tool^1^) and a staff-facing decision support (Decision Support Tool). Children/caregivers completed the screening tool during their visit on an electronic tablet device at the CAC, and screening results were inserted into the subsequent decision support REDCap form where CAC staff documented their decisions and actions. Timing and workflows were determined by individual CACs [see (18)]. All records for these analyses were de-identified. All procedures and a waiver of informed consent were approved by the University of Utah Institutional Review Board.

### Participants

Participants were children and adolescents between 5 and 18 years old visiting a participating CAC during the 2-year period for an initial forensic interview (i.e., interview with the child conducted by a trained professional to elicit facts about maltreatment allegations). We included children with complete responses to the Pediatric Traumatic Stress Screening Tool; we excluded children seen solely for therapy or follow-up, children with a primary language other than English or Spanish, and children whose records were missing date, site of administration, or age.

### Measures

#### Pediatric traumatic stress screening tool

The screening tool was available in caregiver- and youth-report versions in English and Spanish. Caregiver report was recommended for children aged 5–10 years old and self-report for children aged 11–18 years old. Before administering the screening tool, CAC staff recorded child demographics (i.e., age, gender, race, ethnicity) and the reason(s) for the CAC visit on a linked form. The screening tool captured exposure to potentially traumatic events and traumatic stress symptoms; it also included one screening question for suicidality. Potential traumatic exposures and traumatic stress symptoms were assessed with the UCLA PTSD Reaction Index Brief Form (22). Subscales of the Brief Form assess specific domains of PTSD symptoms: intrusion, avoidance, negative alternations in cognitions and mood, and arousal/reactivity. After indicating and describing recent and remote traumatic experiences, respondents rated 11 symptom frequency items on a 5-point scale from 0 “none” to 4 “most” of the time during the past month.^2^ Prior studies of the UCLA Brief Form have found excellent internal consistency (*α* > 0.90) and support for the measure’s clinical utility in discriminating between cases with and without PTSD using a cutoff score of 21 (22). Internal consistency for this study was excellent (*α* = 0.92).

Decision support for the CPM-PTS classified risk for PTSD as high (score ≥ 21), moderate (score 11–20), or low (score ≤ 10). The moderate risk category was added to identify children within this high-risk population who may benefit from psychoeducation, brief “light-touch” interventions, and/or further evaluation. A question from the Patient Health Questionnaire-Adolescent (23, 24) was used to assess risk for suicide and/or self-harm (i.e., thoughts that you would be better off dead or thoughts of hurting yourself). The CPM-PTS Pediatric Traumatic Stress Screening Tool is freely available within the published protocol (16).

#### Decision support tool

The Decision Support Tool guided CAC staff through a three-step process: (1) report any new maltreatment allegations and/or respond to other identified safety concerns, (2) evaluate and respond to suicidality, and (3) provide brief interventions and/or referrals to mental health care (see (18) for more details). At each step, the tool suggested appropriate actions. Staff could choose to take other actions in place of and/or in addition to those suggested. Any positive response to the suicidality screening question prompted CAC staff to administer the Columbia Suicide Severity Rating Scale (25), which classified the level of suicide risk and suggested appropriate response options based on the level of risk (e.g., safety planning, facilitating immediate crisis response) [see (26)].

The Decision Support Tool suggested brief interventions and referral options based on the domain and severity of traumatic stress symptoms, prioritizing sleep problems when present, then symptoms of intrusion and/or hyperarousal/reactivity, and finally symptoms of avoidance and/or negative alterations in cognitions and mood (27–29). Brief interventions suggested for children with elevated sleep problems included the nighttime use of diaphragmatic breathing or guided imagery. Suggested brief interventions for children with elevated symptoms of intrusion and/or hyperarousal/reactivity were daytime use of diaphragmatic breathing, guided imagery, and mindfulness. Lastly, suggested brief interventions for children with elevated symptoms of avoidance and/or negative mood were caregiver-child communication, behavioral activation, and caregiver-child special time. Staff could deliver more than one brief intervention.

The Decision Support Tool also suggested referral options for families. Referral options included follow-up with primary care provider and/or referral to general community mental health services for children categorized as low risk for PTSD and referral to evidence-based trauma treatment (e.g., Trauma-Focused Cognitive-Behavioral Therapy) for those at moderate or high PTSD risk. Staff could also recommend other actions (e.g., follow-up with existing mental health provider). Staff documented their actions and decisions (e.g., delivery of brief intervention, referral to evidence-based trauma therapy) within the Decision Support Tool.

### Analyses

We analyzed data for children seen for an initial forensic interview during the 2-year study period. Screening data were considered complete if at least 10 of 11 questions on the UCLA Brief Form were completed; a missing question was assigned the mean response of completed questions. We first used Pearson’s chi-square tests of independence to examine differences in missing data by child and CAC characteristics (e.g., demographic characteristics, urban vs. rural CAC location) to evaluate potential bias.

We then conducted a set of analyses focused on child characteristics and symptoms. Our outcomes were total traumatic stress symptom score and PTSD risk category. Child characteristics examined were gender, race, ethnicity, age, and reason for CAC visit as indicated by staff. We also described differences in symptoms for children seen in rural vs. urban CACs. We examined descriptive statistics and used Pearson’s chi-square tests and multilevel regression models to examine associations of child characteristics with outcomes. Multilevel analyses were conducted in R using the *lme4* package; other analyses were conducted in SPSS. Multilevel models included child age, gender (cisgender male vs. cisgender female or transgender male^3^), race/ethnicity (non-Hispanic white vs. minoritized group), and concern for sexual abuse (no vs. yes) along with a random effect to account for clustering within CACs. For traumatic stress symptom scores, we conducted linear mixed models fit by restricted maximum likelihood estimation. For PTSD risk category, we conducted binomial generalized linear mixed models fit by maximum likelihood.

Our next set of analyses focused on CAC staff’s decision-making and responses. We described staff delivery of brief interventions and referral decisions recorded in the Decision Support Tool. We used Pearson’s chi-square tests to test for differences in responses by PTSD risk category and suicidality. Binomial generalized linear mixed models were conducted to test associations of child characteristics with delivery of brief interventions and referral decisions. Models included PTSD risk category, age, gender, race/ethnicity, concern for sexual abuse, and a random effect to account for clustering within CACs.

## Results

### Missing data

The CPM-PTS was adopted and administered electronically by 16 CACs. On average, CACs administered the CPM-PTS to 53% of the children they served. Screening rates ranged from 10 to 100% across CACs [see (18) for more information on implementation outcomes and determinants of use]. During the 2-year period of this study, CPM-PTS administration was initiated with 2,569 children. Nine percent of these children (*n* = 219) were excluded from analyses because of missing data (75 missing age; 114 missing symptom data; 30 missing age and symptom data), resulting in an analytic sample of 2,350 children. Missing data were more common among children seen in rural CACs compared to urban CACs (11.5% vs. 6.2%, *χ*^2^ = 22.77, *p* < 0.01) and children aged 5–10 compared to adolescents aged 11–18 (6.4% vs. 3.9%, *χ*^2^ = 7.06, *p* < 0.01). There were no differences in the likelihood of missing data by child gender, race, or ethnicity. Referral decisions were documented for 83% of the children included in analyses (*n* = 1,950).

### Characteristics and symptoms of children completing the CPM

Child characteristics are presented in Table 1. Children were mostly female, white, non-Hispanic, adolescents (*M* = 12.96 years, SD = 3.36), and visiting the CAC for concerns about sexual abuse. As expected, child characteristics varied across sites due to differences in the populations of their catchment area and their criteria for service. Traumatic stress symptom scores ranged from 0 to 44 (*M* = 19.20; SD = 11.60). Close to half of children (45.5%) were categorized as high PTSD risk, and another quarter (26.7%) were at moderate PTSD risk. Suicidality and staff responses to suicide risk among 11–18 year-old youth are described by Shepard et al. (26). See Supplementary file 1 for information about suicidality within the full sample of 5–18 year-olds.

**Table 1:** PTSD risk category and child characteristics (*N* = 2,350).

	*N* (%)				*χ* ^2^
		Risk for PTSD			
	Full sample	Low	Moderate	High	
		653 (27.8)	628 (26.7)	1,069 (45.5)	
Gender					113.78**
Female	1,701 (72.4)	401 (23.6)	418 (24.6)	882 (51.9)	
Male	619 (26.3)	247 (39.9)	200 (32.3)	172 (27.8)	
Transgender male	6 (0.3)	0 (0.0)	2 (33.3)	4 (66.7)	
Unknown^a^	24 (1.0)				
Race					32.56**
American Indian/Alaska Native	63 (2.7)	20 (31.7)	21 (33.3)	22 (34.9)	
Asian	14 (0.6)	3 (21.4)	3 (21.4)	8 (57.1)	
Black/African American	29 (1.2)	11 (37.9)	4 (13.8)	14 (48.3)	
Multiracial	72 (3.1)	16 (22.2)	26 (36.1)	30 (41.7)	
Native Hawaiian/Pacific Islander	30 (1.3)	19 (63.3)	3 (10.0)	8 (26.7)	
Other	15 (0.6)	2 (13.3)	6 (40.0)	7 (46.7)	
White	1,824 (77.6)	499 (27.4)	491 (26.9)	834 (45.7)	
Unknown^a^	303 (12.9)				
Ethnicity					1.74
Hispanic/Latinx	313 (13.3)	80 (25.6)	80 (25.6)	153 (48.9)	
Non-Hispanic/Latinx	2,037 (86.7)	573 (28.1)	548 (26.8)	916 (45.1)	
Age					106.65**
5–10 years old	639 (27.2)	261 (40.8)	190 (29.7)	188 (29.4)	
11–18 years old	1,711 (72.8)	392 (22.9)	438 (25.6)	881 (51.5)	
**Reason for CAC visit** ^b^ **—concern for:**					
Sexual abuse	1,698 (72.3)	430 (25.3)	436 (25.7)	832 (49.0)	32.44**
Physical abuse	402 (17.1)	125 (31.1)	128 (31.8)	149 (37.1)	14.23**
Witnessed domestic violence	150 (6.4)	55 (36.7)	53 (35.3)	42 (28.0)	19.77**
Neglect	107 (4.6)	32 (29.9)	31 (29.0)	44 (41.1)	0.86
Harmful material(s)	91 (3.9)	28 (30.8)	26 (28.6)	37 (40.7)	0.91
Witnessed crime	47 (2.0)	11 (23.4)	15 (31.9)	21 (44.7)	0.82
Other	130 (5.5)	50 (38.5)	32 (24.6)	48 (36.9)	8.10*
Location of CAC visit					25.56**
Urban CAC	1,338 (56.9)	322 (24.1)	356 (26.6)	660 (49.3)	
Rural/frontier CAC	1,012 (43.1)	331 (32.7)	272 (26.9)	409 (40.4)	

a Cases with unknown demographic characteristics were not included in chi-square analyses.

b Multiple reasons for CAC visit could be selected. Analyses compare cases with and without that reason selected.

**p < 0.01; *p < 0.05.

Table 1 shows differences in PTSD risk category by child characteristics. Children who identified as female or transgender male were more likely to be high PTSD risk than those who identified as male, and older children were more likely to be high PTSD risk than younger children. There were significant differences by race, although sample sizes for most groups were small, and no differences by ethnicity. Children seen for concerns about sexual abuse were more likely to be high PTSD risk compared to those without this concern, and those seen for concerns about physical abuse, witnessing domestic violence, or other reasons were less likely to be high PTSD risk than those without these concerns. There were significant differences between children seen in urban and rural CACs; more children seen in urban CACs were categorized as high PTSD risk and fewer children were low risk.

In a mixed linear model accounting for nesting of children within CACs, gender (female or transgender male; *b* = 4.11, *p* < 0.001) and older age (*b* = 0.83, *p* < 0.001) were associated with higher traumatic stress symptom scores; race/ethnicity and concern for sexual abuse were not significantly associated with symptom scores. Similarly, in multilevel logistic regressions predicting PTSD risk category, female/transgender male gender was associated with a greater likelihood of high PTSD risk (odds ratio = 2.30, *p* < 0.001), and older age was associated with greater likelihood of moderate (odds ratio = 1.06, *p* < 0.001) or high (odds ratio = 1.18, *p* < 0.001) PTSD risk. Race/ethnicity and concern for sexual abuse were not significantly associated with the likelihood of moderate or high PTSD risk. Full model results are presented in Supplementary file 2.

### CAC staff decision making

Most children (77.4%) reported at least one elevated symptom (rating ≥ 3 on 0–4 scale), prompting CAC staff to deliver a brief intervention. Brief interventions were delivered to two-thirds (66.0%) of all children, including 85.2% of cases when a brief intervention was recommended by the CPM-PTS decision support tool. Types of brief interventions are shown in Table 2. The most frequent interventions were teaching diaphragmatic breathing, suggesting ways to improve caregiver-child communication, and teaching guided imagery. Approximately half of the children who received a brief intervention received more than one (32% received 2 interventions; 18% received 3 interventions).

**Table 2:** Brief intervention delivery by symptom cluster.

	Full sample (*N* = 2,350)	Elevated sleep symptom(s) (*n* = 1,012; 43%)	Elevated intrusion/reactivity symptom(s) (*n* = 1,493; 64%)	Elevated avoidance/negative mood symptom(s) (*n* = 1,534; 65%)
Any brief intervention	1,551 (66.0)	877 (86.7)	1,274 (85.3)	1,315 (85.7)
Diaphragmatic breathing	729 (31.0)	502 (49.6)	676 (45.3)	609 (39.7)
Guided imagery	500 (21.3)	430 (42.5)	456 (30.5)	447 (29.1)
Mindfulness	265 (11.3)	187 (18.5)	249 (16.7)	230 (15.0)
Caregiver-child communication	572 (24.3)	310 (30.6)	436 (29.2)	572 (37.3)
Behavioral activation	124 (5.3)	70 (6.9)	103 (6.9)	124 (8.1)
Caregiver-child special time	84 (3.6)	45 (4.4)	72 (4.8)	84 (5.5)
Other/unspecified	324 (13.8)	153 (15.1)	200 (13.4)	193 (12.6)

Categories are not exclusive. Children could have more than one domain with elevated symptoms and/or receive more than one brief intervention. Shaded areas indicate brief interventions recommended for each symptom cluster.

Brief interventions were delivered to 22.3% of children at low risk for PTSD, 78.7% of those at moderate risk, and 85.3% of those at high risk. Children seen in rural CACs were less likely to receive an intervention than those in urban CACs (60% vs. 71%). In multilevel analyses, PTSD risk category was the strongest predictor of brief intervention delivery. Relative to those at low risk, children at moderate risk (odds ratio = 16.05, *p* < 0.001) and high risk (odds ratio = 25.40, *p* < 0.001) were substantially more likely to receive an intervention. There was considerable variation between CACs in brief intervention delivery (conditional ICC = 0.23). Child characteristics were not significantly associated with the likelihood of receiving an intervention (see Supplementary file 2).

Referral decisions are shown in Table 3. Among children with documented referral decisions (83%), most received a referral to evidence-based trauma treatment (53.1%) or community mental health services (39.0%). A small proportion were encouraged to follow up with their primary care provider (2.1%), and other options, such as following up with their existing mental health provider, were reported for the remainder (5.8%). Referrals to evidence-based trauma treatment were made for 45.5% of children at low risk for PTSD, 52.2% of those at moderate risk, and 58.4% of those at high risk. Children seen in rural CACs were less likely to receive a referral to evidence-based trauma treatment than those in urban CACs (44% vs. 60%). In multilevel analyses, PTSD risk category was significantly associated with the likelihood of receiving a referral to evidence-based trauma treatment (moderate PTSD risk odds ratio = 1.58, *p* < 0.01; high PTSD risk odds ratio = 2.24, *p* < 0.001). Again, there was considerable variation between CACs in referral decisions (conditional ICC = 0.24). Child characteristics were not significantly associated with referral decisions (see Supplementary file 2).

**Table 3:** Referral decisions and child PTSD risk category (*N* = 1,950).

	*N* (%)				*χ* ^2^
	Follow up with PCP	Community MH	Evidence-based trauma treatment	Other	
Full sample	41 (2.1)	760 (39.0)	1,036 (53.1)	113 (5.8)	
PTSD risk					79.08**
Low	23 (4.3)	219 (40.7)	245 (45.5)	51 (9.5)	
Moderate	12 (2.3)	221 (41.5)	278 (52.2)	22 (4.1)	
High	6 (0.7)	320 (36.4)	513 (58.4)	40 (4.6)	

**p < 0.01.

## Discussion

We examined traumatic stress symptoms and risk for PTSD within a large sample of children seen in Children’s Advocacy Centers for concerns about child maltreatment. Close to half (45.5%) of these children had a high probability of a PTSD diagnosis at the time of the CAC encounter; another quarter described moderate levels of traumatic stress symptoms that are likely to benefit from specific, trauma-focused interventions. Most children (77.4%) reported one or more elevated traumatic stress symptoms. Older children, those who identified as female or transgender male, and those who were visiting the CAC because of concerns about sexual abuse were at elevated risk. Children seen in urban CACs were more likely to be at high risk for PTSD than children seen in rural CACs, perhaps because of variations in service criteria and the types of cases seen in different CACs. For example, child welfare or law enforcement in urban areas with higher caseloads may have more stringent eligibility criteria for CAC services, resulting in urban CACs serving children who experience more severe maltreatment.

CAC staff delivered brief interventions to most children and were especially likely to do so for children at moderate or high risk for PTSD based on screening results. Approximately half of the children who received a brief intervention received more than one intervention. Children seen in rural CACs were less likely to receive a brief intervention than those in urban CACs, and there was substantial variation in intervention delivery between CACs. This variation is likely because of differences between workflows and staff at different CACs. Our prior work has identified staff self-efficacy as a key determinant of CPM-PTS use. Ongoing training and technical assistance is needed to support CPM-PTS use and may be particularly important for supporting staff without clinical training in their delivery of brief interventions (18, 26). Overall, use of brief interventions was high, and our findings suggest that staff without clinical training can screen children for mental health needs and deliver brief interventions successfully.

Most children received a referral to evidence-based trauma treatment or community mental health services, and the likelihood of a referral to evidence-based trauma treatment was greater for those at greater risk for PTSD. Encouragingly, child gender, age, and race/ethnicity were not associated with the likelihood of a referral. Providing staff and families with specific data to drive decision-making may reduce biases in the referral process. Although referral rates were generally high, not all children at high risk for PTSD received a referral to treatment. It is likely that referral decisions are at least in part reflective of families’ preferences. Staff may not make a referral if a family is already receiving services or is not interested in treatment. Use of evidence-based engagement strategies and follow-up with families may help increase treatment engagement (30).

Mental health workforce shortages and limited availability of specialty mental health services are also likely to affect referral decisions. A substantial proportion of children at elevated PTSD risk were referred to general community mental health services rather than evidence-based trauma treatment, and children seen in rural CACs were less likely to be referred to evidence-based trauma treatment than children seen in urban CACs. These findings likely reflect lack of access to therapists trained specifically in evidence-based trauma treatments, especially in rural areas. Although most CACs report access to evidence-based treatments such as Trauma-Focused Cognitive-Behavioral Therapy (15), they may not have sufficient capacity to serve all the children who could benefit. Ongoing efforts to recruit, train, and retain therapists are needed, especially in rural and frontier areas.

### Limitations

Screening rates varied considerably over time and between CACs, with an average screening rate of 53% across CACs during the 2-year period [see (18)]. There may be selection biases affecting who was offered and who completed the CPM-PTS. However, our prior work suggests that variability in screening was driven primarily by CAC workflows and staff self-efficacy, not child or family characteristics (18). In addition, the demographics of our sample are broadly comparable to the population of children served (31), and our analyses accounted for nesting of children within CACs.

CPM-PTS data were entered by different reporters, including caregivers, youth, and CAC staff. Because child age is confounded with reporter, it is possible that our finding of lower symptoms in younger children may be an artifact of using caregiver vs. self-report. Both the caregiver and self-report versions of the full UCLA PTSD Reaction Index and the self-report version of the Brief Form have strong psychometrics; however, the psychometric properties of the caregiver version of the Brief Form have not yet been examined (22, 32, 33). It should also be noted that the CPM-PTS only screens for traumatic stress symptoms and suicidality, and children served by CACs may have other mental health needs not identified in screening. Children classified as high risk for PTSD or experiencing significant impairment should receive a thorough diagnostic assessment by a mental health professional.

Child demographics were entered by staff, and we do not know whether staff asked children/caregivers about their identities and entered the demographic characteristics described or if staff entered responses based only on their perceptions of children’s identities. Most children were identified as non-Hispanic white (75.7%), consistent with state demographics (77.2%) (34). The very limited racial/ethnic diversity and small numbers of children from most racial/ethnic groups limits our ability to identify meaningful differences in this sample. In addition, the ‘reason for CAC visit’ was entered by staff based on the allegation that brought the family to the center and does not indicate whether the allegation was confirmed. It also may not correspond with the descriptions of recent and/or remote traumatic experiences provided by caregivers and youth on the CPM-PTS screening tool.

## Conclusion

CACs are well-positioned to identify children with mental health needs after allegations of sexual abuse and other maltreatment and provide trauma-informed care (17). In a statewide sample of children served by CACs, we found that almost half were experiencing substantial traumatic stress symptoms and likely to meet PTSD diagnostic criteria at the time of their initial CAC visit. CAC staff, most of whom did not have clinical training, were able to use the CPM-PTS, a structured protocol, to administer a screening tool, deliver brief interventions, and make appropriate referrals to mental health treatment. CACs are a critical setting for early identification of children experiencing traumatic stress symptoms following maltreatment and can facilitate timely access to evidence-based treatments.

## Data availability statement

The raw data supporting the conclusions of this article will be made available by the authors, without undue reservation.

## Ethics statement

The studies involving human participants were reviewed and approved by the University of Utah Institutional Review Board. Written informed consent from the participants’ legal guardian/next of kin was not required to participate in this study in accordance with the national legislation and the institutional requirements.

## Author contributions

EM: conceptualization, formal analysis, writing—original draft. KC: conceptualization, methodology, formal analysis, data curation, writing—review and editing. KB: data curation, project administration, writing—review and editing. LS: project administration, writing—review and editing. BK: conceptualization, methodology, writing—review and editing, supervision, funding acquisition. All authors contributed to the article and approved the submitted version.

## Funding

This work was supported by a grant from the National Child Traumatic Stress Initiative (NCTSI), which is part of the Substance Abuse and Mental Health Services Administration (SAMHSA), to BK, Principal Investigator (Grant Number SM080000-1). EM is supported by a grant from the National Institute of Mental Health (MH123729). The REDCap platform at the University of Utah is supported by 8UL1TR000105 from the National Institutes of Health. The content is solely the responsibility of the authors and does not necessarily represent the official views of the National Institutes of Health. Funding sources had no role in study design, execution, analyses, interpretation, or presentation of results.

## Conflict of interest

KC reported that her institution receives financial compensation for expert witness testimony provided in cases of suspected child abuse for which she is subpoenaed to testify.

The remaining authors declare that the research was conducted in the absence of any commercial or financial relationships that could be construed as a potential conflict of interest.

## Publisher’s note

All claims expressed in this article are solely those of the authors and do not necessarily represent those of their affiliated organizations, or those of the publisher, the editors and the reviewers. Any product that may be evaluated in this article, or claim that may be made by its manufacturer, is not guaranteed or endorsed by the publisher.

## Footnotes

^1^In this context, pediatric traumatic stress refers to any traumatic stress experienced by children; it is not specific to medical traumatic stress.

^2^An additional item from the UCLA PTSD Reaction Index assessing distressing dreams was included to identify children who may benefit from brief interventions targeting sleep; this item was not included in scoring of PTSD symptoms.

^3^Transgender male was not included as a separate category because of the small sample size (n = 6). We chose to group transgender males and cisgender females together for analyses because both are marginalized groups relative to cisgender males.
