# Supplementary material for: Traumatic stress symptoms and PTSD risk in children served by Children’s Advocacy Centers
Source: Front Psychiatry. 2023 Jun 29;14:1202085. doi: 10.3389/fpsyt.2023.1202085 (PMC10346839; doi:10.3389/fpsyt.2023.1202085)
Supplement: Supplementary file 1 [file Table_1.DOCX]

**Supplemental File 1**

*Suicidality Results for All Youth*

Suicidality and staff responses to suicide risk for 11-18 year-old youth are described by Shepard and colleagues (2023). This supplemental file includes information about suicidality within the full sample of 5-18 year-olds.

**Analyses**

We examined descriptive statistics and used chi-square tests and multilevel regression analyses to examine associations of child characteristics with suicidality outcomes. Multilevel models included child age, gender (cisgender male vs. cisgender female or transgender male), race/ethnicity (non-Hispanic white vs. minoritized group), and concern for sexual abuse (no vs. yes). We used the *lme4* package in R to conduct binomial generalized linear mixed models fit by maximum likelihood for each outcome (i.e., presence of any suicidal ideation, identification as at high risk for suicide). A small number of youth (n = 13) were missing data on suicidality and excluded from these analyses.

**Results**

Thoughts about suicide/self-harm were endorsed by 39.1% of youth. Based on follow-up questions using the CSSRS, 246 youth were classified as high risk for suicide (10.5% of the full sample; 26.9% of those reporting thoughts about suicide/self-harm). Children seen in urban CACs were more likely to report any ideation than those seen in rural CACs (42.4% vs. 34.8%); the same pattern was found for children at high risk for suicide (11.6% vs. 9.0%). Differences in suicidal ideation and suicide risk by child characteristics are shown in Table 1. Female children, older children, Hispanic/Latinx children, and children seen for concerns about sexual abuse were more likely to report any suicidal ideation and be identified as at high risk for suicide.

In multilevel analyses accounting for nesting within CACs, children who identified as female or transgender male were more likely to report suicidal ideation than those who identified as male, and older children were more likely to report suicidal ideation than younger children (Table 2). Gender and age were also significantly associated with a higher likelihood of being identified as at high risk for suicide (Table 3). Race/ethnicity and concern for sexual abuse were not significantly associated with the likelihood of any ideation or identification as high risk. Referral decisions for youth with any suicidal ideation and youth identified as at high risk for suicide are shown in Table 4.

**Table 1**

*Suicidality and Child Characteristics (N = 2337)*

|  | **Any Thoughts about Suicide/Self-harm** | | **Identified as High Risk for Suicide** | |
| --- | --- | --- | --- | --- |
|  | **N (%)** | **χ^2^** | **N (%)** | **χ^2^** |
| Full Sample | 914 (39.1) |  | 246 (10.5) |  |
| Gender |  | 81.10** |  | 40.88** |
| Female | 749 (44.2) |  | 222 (13.1) |  |
| Male | 152 (24.8) |  | 24 (3.9) |  |
| Transgender male | 6 (100.0) |  | 0 (0.0) |  |
| Race |  | 15.10* |  | 12.11+ |
| American Indian/Alaska Native | 16 (26.2) |  | 2 (3.3) |  |
| Asian | 5 (35.7) |  | 1 (7.1) |  |
| Black/African American | 10 (34.5) |  | 3 (10.3) |  |
| Multiracial | 26 (36.6) |  | 5 (7.0) |  |
| Native Hawaiian/Pacific Islander | 9 (30.0) |  | 2 (6.7) |  |
| Other | 3 (20.0) |  | 0 (0.0) |  |
| White | 705 (38.8) |  | 189 (10.4) |  |
| Unknown | 140 (46.5) |  | 44 (14.6) |  |
| Ethnicity |  | 4.70* |  | 5.92* |
| Hispanic/Latinx | 139 (44.7) |  | 45 (14.5) |  |
| Non-Hispanic/Latinx | 775 (38.3) |  | 201 (9.9) |  |
| Age |  | 138.18** |  | 65.98** |
| 5-10 years old | 124 (19.6) |  | 13 (2.1) |  |
| 11-18 years old | 790 (46.3) |  | 233 (13.7) |  |
| Reason for CAC Visit – Concern for: |  |  |  |  |
| Sexual abuse | 705 (41.7) | 17.41** | 197 (11.7) | 8.28** |
| Physical abuse | 137 (34.3) | 4.61* | 31 (7.8) | 3.88* |
| Witnessed domestic violence | 38 (25.3) | 12.78** | 8 (5.3) | 4.59* |
| Neglect | 41 (38.3) | 0.03 | 11 (10.3) | 0.01 |
| Harmful material(s) | 38 (41.8) | 0.28 | 12 (13.2) | 0.71 |
| Witnessed crime | 18 (38.3) | 0.01 | 5 (10.6) | 0.00 |
| Other | 48 (37.2) | 0.21 | 13 (10.1) | 0.03 |

*Note:* Multiple reasons for CAC visit could be selected. Analyses compare cases with and without that reason selected.

** *p* < .01 * *p* < .05 + *p* < .10

**Table 2**

*Child Characteristics Associated with Report of Any Suicidal Ideation*

|  | **Unstandardized Coefficient (SE)** | **Odds Ratio (95% CI)** |
| --- | --- | --- |
| Gender | 0.61** (0.12) | 1.83 (1.46-2.31) |
| Age | 0.15** (0.01) | 1.16 (1.13-1.19) |
| Race/ethnicity | 0.01 (0.11) | 1.02 (0.82-1.25) |
| Concern for sexual abuse | -0.08 (0.11) | 0.93 (0.74-1.16) |
| *Conditional ICC* | *0.0163* | |

* *p* < .01 ** *p* < .001

**Table 3**

*Child Characteristics Associated with Categorization as High Risk for Suicide*

|  | **Unstandardized Coefficient (SE)** | **Odds Ratio (95% CI)** |
| --- | --- | --- |
| Gender | 0.95** (0.24) | 2.58 (1.62-4.11) |
| Age | 0.19** (0.03) | 1.21 (1.14-1.27) |
| Race/ethnicity | 0.08 (0.17) | 1.08 (0.78-1.49) |
| Concern for sexual abuse | -0.08 (0.19) | 0.92 (0.64-1.32) |
| *Conditional ICC* | *0.0556* | |

* *p* < .01 ** *p* < .001

**Table 4**

*Referral Decisions and Child Symptoms (N = 1950)*

|  | **N (%)** | | | | χ^2^ |
| --- | --- | --- | --- | --- | --- |
|  | **Follow up with PCP** | **Community MH** | **Evidence-based Trauma Treatment** | **Other** |  |
| Full sample | 41 (2.1) | 760 (39.0) | 1036 (53.1) | 113 (5.8) |  |
| Suicide risk |  |  |  |  |  |
| Any ideation | 15 (2.0) | 294 (39.4) | 398 (53.3) | 40 (5.4) | 10.77* |
| High risk for suicide | 4 (1.8) | 92 (42.0) | 108 (49.3) | 15 (6.8) | 4.25 |

** *p* < .01 * *p* < .05
