# Supplementary material for: Traumatic stress symptoms and PTSD risk in children served by Children’s Advocacy Centers
Source: Front Psychiatry. 2023 Jun 29;14:1202085. doi: 10.3389/fpsyt.2023.1202085 (PMC10346839; doi:10.3389/fpsyt.2023.1202085)
Supplement: Supplementary file 2 [file Table_2.DOCX]

**Supplemental File 2**

*Results of Multilevel Models*

Table 1: Child Characteristics Associated with PTSD Symptom Score

Table 2: Child Characteristics Associated with PTSD Risk Category

Table 3: Child Characteristics Associated with Delivery of Brief Intervention

Table 4: Child Characteristics Associated with Referral to Trauma Treatment

**Table 1**

*Child Characteristics Associated with PTSD Symptom Score*

|  | **Unstandardized Coefficient (SE)** | **95% CI** |
| --- | --- | --- |
| Gender | 4.11** (0.56) | 3.02-5.20 |
| Age | 0.83** (0.70) | 0.70-0.97 |
| Race/ethnicity | -0.27 (0.54) | -1.34-0.79 |
| Concern for sexual abuse | 0.60 (0.55) | -0.48-1.69 |
| *Conditional ICC* | *.0234* | |

* *p* < .01 ** *p* < .001

**Table 2**

*Child Characteristics Associated with PTSD Risk Category*

|  | ***Moderate Risk*** | | ***High Risk*** | |
| --- | --- | --- | --- | --- |
|  | **Unstandardized Coefficient (SE)** | **Odds Ratio (95% CI)** | **Unstandardized Coefficient (SE)** | **Odds Ratio (95% CI)** |
| Gender | 0.13 (0.13) | 1.14 (0.88-1.47) | 0.83** (0.13) | 2.30 (1.78-2.97) |
| Age | 0.06** (0.02) | 1.06 (1.03-1.10) | 0.16** (0.02) | 1.18 (1.14-1.22) |
| Race/ethnicity | -0.07 (0.14) | 0.93 (0.71-1.23) | -0.09 (0.13) | 0.92 (0.71-1.18) |
| Concern for sexual abuse | 0.003 (0.14) | 1.00 (0.77-1.31) | 0.10 (0.13) | 1.11 (0.86-1.43) |
| *Conditional ICC* | *0.0429* | | *.0307* | |

* *p* < .01 ** *p* < .001

**Table 3**

*Child Characteristics Associated with Delivery of Brief Intervention*

|  | **Unstandardized Coefficient (SE)** | **Odds Ratio (95% CI)** |
| --- | --- | --- |
| Gender | 0.04 (0.14) | 1.04 (0.79-1.37) |
| Age | -0.004 (0.02) | 1.00 (0.96-1.03) |
| Race/ethnicity | -0.14 (0.14) | 0.87 (0.66-1.14) |
| Concern for sexual abuse | 0.22 (0.14) | 1.25 (0.95-1.64) |
| Moderate PTSD risk | 2.78** (0.15) | 16.05 (11.95-21.55) |
| High PTSD risk | 3.23** (0.15) | 25.40 (19.00-33.97) |
| *Conditional ICC* | *0.2308* | |

* *p* < .01 ** *p* < .001

**Table 4**

*Child Characteristics Associated with Referral to Trauma Treatment*

|  | **Unstandardized Coefficient (SE)** | **Odds Ratio (95% CI)** |
| --- | --- | --- |
| Gender | 0.14 (0.13) | 1.15 (0.88-1.49) |
| Age | -0.02 (0.02) | 0.98 (0.95-1.01) |
| Race/ethnicity | -0.14 (0.13) | 0.87 (0.68-1.12) |
| Concern for sexual abuse | 0.20 (0.13) | 1.23 (0.95-1.58) |
| Moderate PTSD risk | 0.45* (0.14) | 1.58 (1.19-2.09) |
| High PTSD risk | 0.81** (0.13) | 2.24 (1.71-2.93) |
| *Conditional ICC* | *0.2358* | |

* *p* < .01 ** *p* < .001
